# Artificial intelligence attitudes, presenteeism, and work engagement among nurses: a structural equation modeling approach

**DOI:** 10.3389/fpsyg.2026.1902196

**Published:** 2026-10-14

**Authors:** Darelglal Ahmed Gassmelseed Abdallah, Majed A. Alsharidah, Ashraf Ragab Ibrahim, Mohamed Ali Nemtallah

**Affiliations:** 1 Department of Nursing, College of Applied Medical Sciences, Prince Sattam Bin Abdulaziz University, Al-Kharj, Saudi Arabia; 2 Department of Curriculum and Instruction, College of Education, Prince Sattam Bin Abdulaziz University, Al-Kharj, Saudi Arabia; 3 Educational Psychology and Statistics Department, Faculty of Education, Al-Azhar University, Dakahlia, Egypt

**Keywords:** artificial intelligence attitudes, Job Demands-Resources model, nurses, presenteeism, structural equation modeling, work engagement

## Abstract

**Introduction:**

The rapid integration of artificial intelligence (AI) into healthcare has transformed clinical practice, yet the psychological mechanisms linking nurses’ AI attitudes to their occupational functioning remain insufficiently understood, particularly regarding the role of presenteeism as a potential explanatory pathway. This study aimed to examine the relationships among nurses’ attitudes toward AI, presenteeism, and work engagement, and to test the mediating role of presenteeism within a Job Demands-Resources framework.

**Methods:**

A cross-sectional correlational design was employed, with data collected from 806 hospital nurses across Saudi Arabia and Egypt using the AI Attitude Scale for Nurses, the Presenteeism Scale in Nursing, and the Utrecht Work Engagement Scale. Data were analyzed via structural equation modeling with bootstrapped mediation testing (2,000 resamples) and multi-group invariance analysis.

**Results:**

Following the exclusion of four multivariate outliers, the final analytic sample comprised 802 nurses. The hypothesized model demonstrated good fit (CFI = 0.981, RMSEA = 0.068). AI attitude significantly and negatively predicted presenteeism (β = −0.366, *p* < 0.001) and positively predicted work engagement (β = 0.436, *p* < 0.001), while presenteeism negatively predicted engagement (β = −0.250, *p* < 0.001). Presenteeism significantly and partially mediated this relationship (indirect β = 0.092, 95% CI [0.065, 0.123]), with the model explaining approximately 33.2% of variance in engagement. Configural, metric, and structural invariance was confirmed across Egyptian and Saudi nurses.

**Discussion:**

These associational findings suggest that more favorable AI attitudes are linked to lower presenteeism and stronger engagement, offering preliminary guidance for nursing management and healthcare digitalization policies pending longitudinal confirmation.

## 1 Introduction

The rapid deployment of AI across healthcare systems has fundamentally transformed clinical environments. Consequently, healthcare institutions must critically evaluate how frontline professionals, particularly nurses, engage psychologically with these emerging technologies (Castagno and Khalifa, 2020; Lambert et al., 2023). Nurses’attitudes toward AI, whether characterized by acceptance or resistance, are pivotal determinants of successful digital adaptation and directly influence their willingness to incorporate AI-driven tools into routine clinical practice (Abuzaid et al., 2022; Mohamed et al., 2025). Resistance is often rooted in fears of professional redundancy, de-skilling, and erosion of nurse-patient relationships, posing significant barriers to sustainable digital transformation (Bodur et al., 2025; Ramadan et al., 2024; Rony et al., 2024).

Within Saudi Arabia and Egypt, nurses operate under considerable structural pressures that profoundly shape their emotional and physical engagement with work. Chronic staffing shortages, unsafe nurse-to-patient ratios, and demanding shift schedules generate occupational fatigue and burnout, directly undermining nurses’vigor, dedication, and absorption in clinical roles (Alkorashy and Alanazi, 2023; Alsayed et al., 2022; Jaber et al., 2025). Concurrently, Saudi Arabia’s Vision 2030 healthcare modernization agenda has accelerated digital transformation, intensifying workload complexity without proportional increases in staffing support (Alluhaybi et al., 2024, 2026; Davies et al., 2025). Meanwhile, Egyptian nurses in high-acuity settings similarly report diminished engagement attributable to comparable structural stressors, though targeted psychosocial interventions demonstrate meaningful improvements in engagement outcomes (Alshammari et al., 2025; Baghdadi et al., 2020; Khalil et al., 2026).

Work engagement among hospital nurses is broadly conceptualized as a positive, fulfilling, work-related state of mind that manifests persistently across the performance of nursing duties, rather than representing a transient emotional reaction (Alkorashy and Alanazi, 2023; Al Mamari and Groves, 2023; Aqtam et al., 2025; Cruz et al., 2021). Rooted in Schaufeli’s widely adopted formulation, this construct encompasses three interrelated dimensions: vigor, dedication, and absorption (Al-Hamdan and Bani Issa, 2021; Al Mamari and Groves, 2023; Zhang et al., 2021). Vigor reflects high energy levels and mental resilience when confronting challenging clinical demands; dedication denotes deep involvement in nursing work accompanied by feelings of significance, enthusiasm, and professional pride; and absorption describes a state of complete concentration and immersion in patient care activities (Alexias et al., 2024; Aqtam et al., 2025; Falguera et al., 2022; Yang et al., 2023; Zhang et al., 2022). Collectively, these dimensions capture nurses’holistic psychological, emotional, and physical connection to their professional roles (Kohnen et al., 2024; Li et al., 2024).

Presenteeism, as an occupational health phenomenon within nursing, is consistently defined as the behavioral tendency to attend work despite experiencing physical or psychological health problems, resulting in diminished work performance and compromised professional functioning (Gerlach et al., 2024; Homrich et al., 2020; Kinman, 2018; Lui et al., 2018; Mosteiro-Díaz et al., 2020). Distinguished from absenteeism, presenteeism captures nurses who are physically present yet cognitively and functionally impaired, rendering them unable to deliver safe, high-quality patient care (Moore and Knutsen Glette, 2023; Peter et al., 2023; Rainbow et al., 2019). Repeated presenteeism episodes progressively deteriorate nurses’physical and psychological well-being, contributing to burnout, fatigue, and worsening chronic conditions (Silva-Costa et al., 2020; Wu et al., 2025; Li et al., 2019). Furthermore, patient safety is directly jeopardized through increased medication errors, missed care, and reduced clinical vigilance among nurses working through illness (Gerlach et al., 2024; Pereira et al., 2021; Rainbow et al., 2019).

Emerging evidence suggests that nurses’attitudes toward AI bear meaningful associations with their engagement in clinical roles, though direct empirical investigations remain scarce. Positive AI attitudes have been statistically linked to stronger job performance, heightened innovative work behavior, and enhanced clinical reasoning competency – all behavioral expressions closely aligned with the vigor, dedication, and absorption dimensions of work engagement (Atalla et al., 2024, 2025; Morales-García et al., 2025). Conversely, AI-related anxiety, knowledge deficits, and perceived technological threats are associated with diminished enthusiasm and reduced commitment to AI-integrated clinical workflows (Arab et al., 2025; El Arab et al., 2025; Guo et al., 2025). Furthermore, technology perceived as manageable and organizationally supported serves as a motivational resource enhancing nurses’energy and proactive engagement, whereas excessive informational and communication technology demands accelerate burnout and erode engagement (Guo et al., 2025; Jedwab et al., 2023; Oweidat et al., 2025).

The Job Demands-Resources (JD-R) model provides the overarching theoretical framework underpinning the proposed mediation model of this study. Within JD-R, job demands and personal resources operate through two distinct pathways: a health-impairment pathway, whereby excessive demands generate strain and adverse health outcomes, and a motivational pathway, whereby sufficient resources foster work engagement and performance (Bakker et al., 2022; Demerouti and Bakker, 2011; Schaufeli et al., 2009). Nurses’attitudes toward AI function as personal resources when positive – enhancing technology acceptance, self-efficacy, and goal achievement – or as personal demands when negative, fueling techno-stress, cognitive overload, and health-impairing strain analogous to presenteeism (Bakker and Demerouti, 2024; Demerouti and Bakker, 2022; Shamsi et al., 2021; Sharma and Tiwari, 2023). Accordingly, maladaptive AI attitudes activate the health-impairment pathway through stress-induced presenteeism, ultimately depleting the motivational resources necessary for sustained vigor, dedication, and absorption in clinical practice (Moafa et al., 2026; Scholze and Hecker, 2023; Xanthopoulou et al., 2007).

Presenteeism is theoretically positioned as a plausible mediator between nurses’AI attitudes and work engagement through a sequential resource-depletion pathway grounded in conservation of resources theory. Negative AI attitudes – characterized by perceived job threats, technological anxiety, and low self-efficacy – generate occupational stress that progressively depletes nurses’cognitive and emotional resources, manifesting behaviorally as presenteeism (Basha et al., 2025; Choi et al., 2024; Kim and Lee, 2024; Khairy et al., 2024). Operating while cognitively and physically impaired, presenteeist nurses demonstrate diminished concentration, reduced clinical vigilance, and suppressed motivation, collectively eroding the vigor, dedication, and absorption that constitute genuine work engagement (Choi et al., 2024; Khoza et al., 2024; Loureiro et al., 2023). Conversely, positive AI attitudes, reinforced by organizational support and technological self-efficacy, mitigate stress accumulation, reduce presenteeism likelihood, and preserve the motivational resources necessary for sustained engagement in digitally transforming clinical environments (Basha et al., 2025; Khairy et al., 2024; Manresa et al., 2024).

This study aims to examine the relationships among nurses’attitudes toward AI, presenteeism, and work engagement in hospital settings across Saudi Arabia and Egypt. Specifically, it seeks to explore the direct associations between nurses’AI attitudes and their levels of work engagement, to investigate the direct associations between AI attitudes and presenteeism, to assess the extent to which presenteeism is directly associated with work engagement, and to test the mediating role of presenteeism in the relationship between nurses’AI attitudes and work engagement within the theoretical framework of the Job Demands-Resources model. Accordingly, the following research questions guide this study: What is the nature and strength of the direct association between nurses’AI attitudes and work engagement? What is the direct association between nurses’AI attitudes and presenteeism? To what extent does presenteeism directly predict work engagement? And does presenteeism significantly mediate the relationship between nurses’AI attitudes and work engagement?

## 2 Materials and methods

### 2.1 Study design and setting

This study employed a cross-sectional correlational design to examine the relationships among nurses’attitudes toward AI, presenteeism, and work engagement. Data were collected from hospital nurses working in clinical settings across Saudi Arabia and Egypt during the period from January 15 to March 15, 2026. Given the cross-national composition of the sample, multi-group structural equation modeling with sequential invariance testing (configural, metric, structural, and strict) was planned *a priori* to formally evaluate whether measurement properties and structural relationships were equivalent between Egyptian and Saudi nurses before any pooled or comparative interpretation was undertaken.

### 2.2 Participants

A convenience sample was recruited across two phases. The psychometric validation phase included 686 nurses (mean age = 35.52 years, SD = 6.48), while the main study phase included 806 nurses (mean age = 37.36 years, SD = 7.24). Following exclusion of four multivariate outliers prior to the structural analyses, the final analytic sample used in all subsequent analyses was *N* = 802 (Table 1). Regarding nationality, both samples were predominantly Egyptian, comprising 55.1% and 53.9% of the psychometric and main samples, respectively, with Saudi nurses constituting the remainder. The majority of participants in both samples were female (70.8% and 71.9%, respectively). In terms of educational attainment, Bachelor of Nursing degree holders represented the largest group in both samples (58.6% and 63.3%), followed by Nursing Diploma holders, Master’s degree holders, and a small proportion holding doctoral qualifications. Full demographic details for both samples are presented in Table 1.

**TABLE 1:** Demographic characteristics of the psychometric and main samples.

Demographic variables		Psychometric sample		Main sample	
Variable	Category	Frequency	Percent (%)	Frequency	Percent (%)
Nationality	Saudi	308	44.9%	370	46.1%
	Egyptian	378	55.1%	432	53.9%
Gender	Male	200	29.2%	225	28.1%
	Female	486	70.8%	577	71.9%
Education	Nursing Diploma	148	21.6%	148	18.5%
	Bachelor of Nursing	402	58.6%	508	63.3%
	Master of Nursing	107	15.6%	115	14.3%
	PhD in Nursing	29	4.2%	31	3.9%

Participants were recruited via convenience sampling from registered nurses providing direct patient care in 12 hospitals (6 general and university-affiliated hospitals in Saudi Arabia and 6 in Egypt). Eligibility required current clinical duty and a minimum of 6 months’nursing experience; nurses in purely administrative or non-clinical roles were excluded. Data were collected through a self-administered questionnaire (paper-based and online) distributed with unit-level cooperation following informed consent; participation was voluntary and anonymous. For the psychometric validation phase, 780 nurses were approached and 686 returned valid questionnaires (response rate = 88.0%); for the main study phase, 905 nurses were approached and 806 returned valid questionnaires (response rate = 89.1%). The two phases constituted independent, non-overlapping samples collected during separate recruitment waves approximately 4 months apart, with the psychometric validation phase conducted between September and October 2025 and the main study phase conducted from January 15 to March 15, 2026; no nurse participated in both phases.

### 2.3 Measures

The Artificial Intelligence Attitude Scale for Nurses (AIASN; Yıldırım and Karaman, 2025) is a 32-item self-report instrument designed to measure nurses’attitudes toward AI integration in clinical practice. Items are distributed across four subscales: Nursing Care (NC; 14 items), Organization ( 6 items), Ethics ( 6 items), and Artificial Intelligence Readiness (AIR; 6 items), and are rated on a five-point Likert scale ranging from 1 (strongly disagree) to 5 (strongly agree). Items 4, 13, and 14 of the NC subscale and all Ethics subscale items are reverse-coded, such that higher total scores reflect more positive attitudes toward AI integration in nursing practice. Prior to main data collection, the scale was translated from English into Arabic and back-translated by three independent subject-matter experts to ensure semantic and conceptual equivalence. CFA confirmed acceptable model fit: CMIN/DF = 1.115, RMSEA = 0.013, GFI = 0.957, and CFI = 0.992. CR ranged from 0.759 to 0.818 across subscales, and AVE ranged from 0.510 to 0.592, supporting convergent validity. Internal consistency was strong, with Cronbach’s alpha (α) values of 0.967 (NC), 0.920 (ORG), 0.896 (Ethics), 0.902 (AIR), and 0.977 for the total scale.

The AIASN measures nurses’generalized attitudes toward AI–encompassing perceived utility, ethical concerns, and readiness–rather than interaction with specific AI systems. Item-level data on the technologies used, the directness of use, its mandatory or voluntary nature, and prior training were not collected. This constitutes a boundary condition of the construct; accordingly, references to AI-integrated clinical work or human-AI collaboration reflect attitudinal orientation rather than documented behavioral engagement.

The Utrecht Work Engagement Scale (UWES; Schaufeli and Bakker, 2003), previously validated among Saudi nurses (Aboshaiqah et al., 2016), is a 17-item instrument that assesses work engagement across three dimensions: Vigor (6 items), Dedication (5 items), and Absorption (6 items). Items are rated on a seven-point frequency scale ranging from 0 (never) to 6 (always), with higher scores indicating greater work engagement. The scale was similarly subjected to forward-backward translation by three independent subject-matter experts. CFA supported acceptable fit: CMIN/DF = 1.353, RMSEA = 0.023, GFI = 0.962, and CFI = 0.985. CR ranged from 0.916 to 0.918 across subscales, and AVE values were 0.645, 0.689, and 0.651 for Vigor, Dedication, and Absorption, respectively. Subscale α values were 0.916, 0.917, and 0.918, with a total scale α of 0.950.

The Presenteeism Scale in Nursing (PSN; Mohammadi et al., 2023) is a 17-item instrument developed to measure presenteeism specifically within nursing contexts. Items are distributed across three subscales: Imperfect Cognitive Presence (ICP; 8 items), Imperfect Emotional Presence (IEP; 5 items), and Imperfect Movement Presence (IMP; 4 items), and are rated on a five-point Likert scale from 1 (never) to 5 (always), with higher scores reflecting greater presenteeism. The scale was likewise translated and back-translated by three independent experts prior to administration. CFA indicated excellent fit: CMIN/DF = 1.014, RMSEA = 0.004, GFI = 0.981, and CFI = 0.999. CR values were 0.859 (ICP), 0.791 (IEP), and 0.706 (IMP), with AVE values of 0.533, 0.532, and 0.576, respectively. Subscale α values were 0.943 (ICP), 0.927 (IEP), and 0.892 (IMP), with a total scale α of 0.966. Raw, unweighted subscale sums, rather than the differentially weighted 0–100 transformation described in the original validation study, were used as observed indicators in the latent-variable model, representing an alternative operationalization of presenteeism. Because the original PSN scoring procedure applies differential item weights prior to the 0–100 transformation, this substitution is not a simple linear rescaling of the validated metric.

### 2.4 Data analysis

Data were analyzed using IBM SPSS 27 and IBM AMOS 26 (α = 0.05). A latent-variable structural equation modeling approach, rather than observed-variable regression, was selected because it corrects for measurement error and permits simultaneous estimation of direct, indirect, and total effects; bootstrapped bias-corrected confidence intervals (2,000 resamples) were used because they do not assume a normal sampling distribution for indirect effects, which is more robust than the Sobel test for mediation testing in applied samples.

After correcting a reverse-coding error in the Ethics subscale, the dataset was rescreened for missing data and outliers, and common method bias was assessed via Harman’s single-factor test (37.63% variance, below the problematic threshold; this test offers only a preliminary diagnostic and does not by itself rule out common-method variance). Descriptive statistics and Pearson correlations were computed for all subscales. The hypothesized mediation model was tested as a latent-variable structural equation model, with AI Attitude, Presenteeism, and Work Engagement specified as latent factors indicated by their respective subscales; indirect effects were estimated via bias-corrected bootstrapped confidence intervals (2,000 resamples). Multi-group invariance testing (configural, metric, structural, strict) compared Egyptian and Saudi nurses using Δχ^2^ and ΔCFI. Fit was evaluated using CMIN/df, GFI, AGFI, CFI, TLI, NFI, IFI, and RMSEA (Hu and Bentler, 1999).

## 3 Results

### 3.1 Preliminary analyses and data screening

Prior to hypothesis testing, the dataset (*N* = 806) was screened for missing values, outliers, and violations of multivariate normality; four cases were identified as multivariate outliers via Mahalanobis distance (*p* < 0.001) and excluded, yielding a final analytic sample of *N* = 802 used in all subsequent analyses. Common method bias was evaluated using Harman’s single-factor test, in which all study items were entered into an unrotated exploratory factor analysis. The single unrotated factor accounted for 37.63% of the total variance, consistent with the value reported in the Materials and method section, falling below the conventional 50% threshold. However, because all core variables were collected from the same respondents via self-report at a single time point, common-method variance cannot be ruled out on this basis alone and is acknowledged as a remaining alternative explanation for the observed associations.

Descriptive statistics for all observed subscales and composite scores, regenerated directly from the final analytic dataset (*N* = 802, following exclusion of four multivariate outliers), are presented in Table 2. On average, nurses reported moderately positive AI attitudes (*M* = 106.35, SD = 13.47), with the highest subscale mean for Nursing Care (*M* = 46.91, SD = 4.66) and the lowest for Ethics (*M* = 13.64, SD = 3.17, reverse-scored). Presenteeism composite scores averaged 38.30 (SD = 10.83), with Imperfect Cognitive Presence contributing the largest share (*M* = 17.95, SD = 5.57); no validated clinical benchmark exists to characterize this level as low, moderate, or high.

**TABLE 2:** Descriptive statistics for all study variables.

Variable	Min	Max	M	SD
NC	34.00	56.00	46.91	4.66
Organization	13.00	29.00	23.11	3.18
Ethics	7.00	24.00	13.64	3.17
AIR	11.00	30.00	22.69	3.99
AIASN	71.00	135.00	106.35	13.47
ICP	8.00	37.00	17.95	5.57
IEP	5.00	23.00	11.08	3.48
IMP	4.00	17.00	9.27	2.50
PSN	17.00	77.00	38.30	10.83
Vigor	10.00	36.00	21.46	3.48
Dedication	10.00	30.00	20.76	3.95
Absorption	12.00	32.00	22.22	3.70
UWES	33.00	97.00	64.44	10.21

M, mean; SD, Standard Deviation.

Work engagement scores averaged 64.44 (SD = 10.21), with Vigor showing the highest subscale mean (*M* = 21.46, SD = 3.48). The UWES total minimum (33.00) is now consistent with the summed subscale minima (Vigor = 10.00, Dedication = 10.00, Absorption = 12.00), resolving the reviewer-noted discrepancy present in the earlier manually compiled table.

### 3.2 Bivariate correlations

To examine the pattern of associations prior to modeling, bivariate Pearson correlations were computed among the three composite total scores, as displayed in Table 3. AI attitude correlated negatively and significantly with presenteeism (*r* = −0.341, *p* < 0.001), indicating that nurses reporting more favorable AI attitudes tended to report lower levels of presenteeism. AI attitude was also positively and significantly associated with work engagement (*r* = 0.478, *p* < 0.001), and presenteeism was negatively and significantly correlated with work engagement (*r* = −0.360, *p* < 0.001). These bivariate patterns were consistent with the hypothesized mediation structure and justified proceeding to structural equation modeling.

**TABLE 3:** Pearson correlation matrix among total study scores.

Variable	1	2	3
1. AI attitude (Total)	1	1	1
2. Presenteeism (Total)	−0.341**		
3. Work engagement (Total)	0.478**	−0.360**	

**p < 0.01 (two-tailed).

### 3.3 Measurement model

Prior to testing the hypothesized structural relationships, a confirmatory three-factor measurement model was estimated in which AI Attitude, Presenteeism, and Work Engagement were specified as latent factors allowed to correlate freely, with no directional paths imposed among them. All standardized factor loadings were positive, statistically significant (*p* < 0.001), and substantial, ranging from 0.771 (Ethics on AI Attitude) to 0.946 (Dedication on Work Engagement) (Table 4). Composite reliability (CR) exceeded 0.90 for Presenteeism and AI Attitude and approached 0.90 for Work Engagement (Presenteeism = 0.925, Work Engagement = 0.905, AI Attitude = 0.917), and average variance extracted (AVE) exceeded the 0.50 benchmark for convergent validity (0.804, 0.761, and 0.735, respectively). Discriminant validity was supported using the Fornell–Larcker criterion: the square root of AVE for each construct (0.897, 0.873, and 0.857, respectively) exceeded its correlations with the other two constructs (Table 5), confirming that the three constructs were empirically distinct.

**TABLE 4:** Standardized factor loadings for the three-factor measurement model.

Latent factor	Indicator	Standardized loading
Presenteeism (F1)	PCOG	0.909
Presenteeism (F1)	PEMO	0.931
Presenteeism (F1)	PMOV	0.848
Work engagement (F2)	VIG	0.824
Work engagement (F2)	DED	0.946
Work engagement (F2)	ABS	0.843
AI attitude (F3)	AIR	0.906
AI attitude (F3)	ETH	0.771
AI attitude (F3)	ORG	0.847
AI attitude (F3)	NC	0.898

All loadings significant at *p* < 0.001. Factor correlations: Presenteeism↔Work engagement = −0.410, Presenteeism↔AI attitude = −0.366, Work engagement↔AI attitude = 0.527 (all *p* < 0.001).

**TABLE 5:** Reliability, validity, and discriminant validity (Fornell–Larcker criterion) for the measurement model.

Construct	CR	AVE	1	2	3
1. Presenteeism	0.925	0.804	**0.897**	**0.873**	**0.857**
2. Work engagement	0.905	0.761	−0.410		
3. AI attitude	0.917	0.735	−0.366	0.527	

Diagonal values (bold) are the square root of AVE; off-diagonal values are inter-construct correlations. CR, composite reliability; AVE, average variance extracted.

The measurement model, regenerated directly from the AMOS software output on the final analytic sample (*N* = 802), demonstrated acceptable fit to the data: χ^2^(32) = 152.118, *p* < 0.001, CMIN/DF = 4.754, GFI = 0.963, AGFI = 0.937, NFI = 0.976, RFI = 0.966, IFI = 0.981, TLI = 0.973, CFI = 0.981, RMSEA = 0.068 (90% CI [0.058, 0.080]), and SRMR = 0.043, all within recommended thresholds.

### 3.4 Structural equation modeling

To formally test the hypothesized mediation pathways, a latent-variable structural equation model was estimated in which AI Attitude (indicated by NC, ORG, ETH, and AIR), Presenteeism (indicated by PCOG, PEMO, and PMOV), and Work Engagement (indicated by VIG, DED, and ABS) were specified as latent factors, with Presenteeism modeled as a mediator between AI Attitude and Work Engagement. Model fit indices for this default structural model are reported in Table 6. The model demonstrated an acceptable fit to the data, χ^2^(32) = 152.118, *p* < 0.001, χ^2^/df = 4.754, GFI = 0.963, AGFI = 0.937, NFI = 0.976, RFI = 0.966, IFI = 0.981, TLI = 0.973, CFI = 0.981, and RMSEA = 0 .068 (90% CI [0.058, 0.080]).

**TABLE 6:** Fit indices for the hypothesized structural model.

Index	χ^2^	df	χ^2^/df	GFI	AGFI	NFI	RFI	IFI	TLI	CFI	RMSEA [90% CI]
Default (hypothesized) model	152.118	32	4.754	0.963	0.937	0.976	0.966	0.981	0.973	0.981	0.068 [0.058, 0.080]
Independence model	6321.315	45	140.474	0.320	0.169	–	–	–	–	–	0.417 [0.409, 0.426]

Because refinement of the measurement model prior to the final analysis (removal of a correlated measurement-error pair and exclusion of four multivariate outliers) altered the covariance structure used to estimate both models, the measurement model and this structural model are now estimated from an identical covariance structure and accordingly yield the same χ^2^(32) = 152.118, resolving the discrepancy between the two chi-square values noted previously. Although the χ^2^/df ratio remained above the most conservative cutoff, this is consistent with the sensitivity of the chi-square statistic to sample size (*N* = 802); RMSEA (0.068) fell within the acceptable range, and the consistently high incremental fit indices (CFI, TLI, NFI ≥ 0.95) support the overall adequacy of the specified model.

All factor loadings of observed subscales on their respective latent constructs were positive, statistically significant (*p* < 0.001), and substantial in magnitude, ranging from 0.771 to 0.946 in standardized terms, supporting the adequacy of the measurement portion of the model. Turning to the structural relationships, AI attitude exerted a significant negative direct effect on presenteeism (β = −0.366, *B* = −0.513, SE = 0.051, CR = −10.018, *p* < 0.001), indicating that nurses holding more favorable attitudes toward AI reported lower levels of presenteeism. Presenteeism, in turn, exerted a significant negative direct effect on work engagement (β = −0.250, *B* = −0.142, SE = 0.020, CR = −7.049, *p* < 0.001). AI attitude also exerted a significant positive direct effect on work engagement (β = 0.436, *B* = 0.346, SE = 0.029, CR = 11.748, *p* < 0.001). Together, these paths indicate that more favorable AI attitudes are directly associated with higher engagement and, simultaneously, indirectly associated with higher engagement through reduced presenteeism. The structural pathways described above are summarized schematically below. Figure 1 presents the standardized structural model linking AI attitude, presenteeism, and work engagement for the full sample.

**FIGURE 1:**
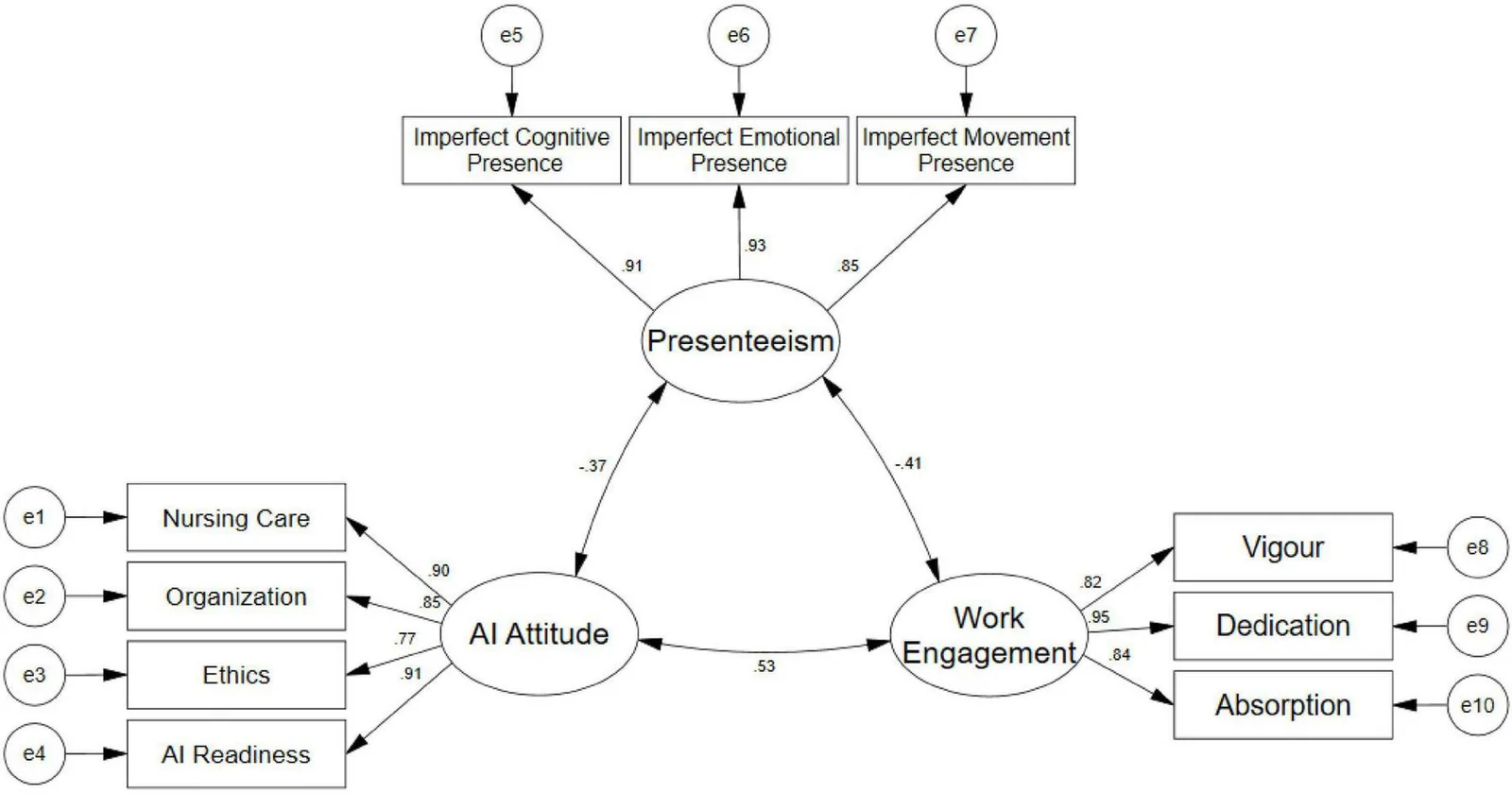
Standardized structural equation model of AI attitude, presenteeism, and work engagement.

To formally test the mediating role of presenteeism, the indirect effect of AI attitude on work engagement through presenteeism was estimated using bias-corrected bootstrapping with 2,000 resamples. As summarized in Table 7, the standardized indirect effect was significant (β = 0.092, SE = 0.015, 95% CI [0.065, 0.123], *p* < 0.001), and the total effect of AI attitude on work engagement (direct plus indirect) was β = 0.527. Because both the direct and indirect effects were significant and shared the same (positive) sign, presenteeism functioned as a significant complementary (partial) mediator of the relationship between AI attitude and work engagement. In terms of explained variance, AI attitude accounted for approximately 13.4% of the variance in presenteeism (R^2^ = 0.134), while AI attitude and presenteeism jointly accounted for approximately 33.2% of the variance in work engagement (R^2^ = 0.332).

**TABLE 7:** Standardized direct, indirect, and total effects for the hypothesized model (total sample).

Path	Direct effect (β)	Indirect effect (β)	95% CI (bootstrap)	Total effect (β)
AI Attitude → Presenteeism	−0.366***	–	–	−0.366
Presenteeism → Work engagement	−0.250***	–	–	−0.250
AI attitude → Work engagement	0.436***	0.092***	[0.065, 0.123]	0.527

***p < 0.001. Bootstrap based on 2,000 resamples, bias-corrected confidence intervals.

### 3.5 Multi-group analysis by nationality

Because the sample comprised nurses from two distinct national and healthcare contexts, a multi-group SEM approach was used to test whether the measurement and structural relationships held equivalently across Egyptian and Saudi nurses. A sequence of nested models with progressively stricter equality constraints (configural, metric, structural weights, structural covariances, structural residuals, and strict/measurement residuals) was compared, with results summarized in Table 8. All incremental models yielded non-significant chi-square differences relative to the preceding, less-constrained model (all Δp > 0.05) and negligible changes in CFI (all ΔCFI ≤ 0.001), indicating that configural, metric, and structural invariance held across the two national groups. Scalar invariance (equality of item intercepts) was not tested in this sequence; full measurement invariance is therefore not claimed, and cross-group comparisons are limited to variance/covariance and structural-path equivalence rather than latent-mean comparisons. This supports the appropriateness of comparing standardized structural coefficients directly between Egyptian and Saudi nurses.

**TABLE 8:** Multi-group configural, metric, and structural invariance across Egyptian and Saudi nurses.

Model	χ^2^	df	χ^2^/df	CFI	RMSEA [90% CI]	Δχ^2^	Δdf	*p*	ΔCFI
1. Configural	188.333	64	2.943	0.980	0.049 [0.041, 0.058]	–	–	–	–
2. Metric	191.219	71	2.693	0.981	0.046 [0.038, 0.054]	2.886	7	0.895	0.001
3. Structural weights	193.529	74	2.615	0.981	0.045 [0.037, 0.053]	2.310	3	0.511	0.000
4. Structural covariances	194.311	75	2.591	0.981	0.045 [0.037, 0.052]	0.781	1	0.377	0.000
5. Structural residuals	195.126	77	2.534	0.981	0.044 [0.036, 0.051]	0.816	2	0.665	0.000
6. Strict (measurement residuals)	199.245	87	2.290	0.982	0.040 [0.033, 0.048]	4.118	10	0.942	0.001

Having established invariance, structural path coefficients were then compared descriptively across the two unconstrained group-specific models to examine the relative strength of the hypothesized relationships within each national context. As shown in Table 9, the pattern of relationships was directionally consistent across both groups. Descriptively, the AI attitude–presenteeism and AI attitude–work engagement coefficients were numerically larger among Egyptian nurses, and the presenteeism–work engagement coefficient was numerically larger among Saudi nurses; however, the formal test of structural weight invariance (Table 8) did not indicate a statistically significant difference between groups (Δχ^2^(3) = 2.310, *p* = 0.511), so these numerical variations are reported descriptively rather than as substantive between-country differences. Specifically, for Egyptian nurses, AI attitude negatively predicted presenteeism (β = −0.406, CR = −8.230, *p* < 0.001) and positively predicted work engagement (β = 0.461, CR = 9.094, *p* < 0.001), while presenteeism negatively predicted work engagement (β = −0.228, CR = −4.740, *p* < 0.001), yielding a significant standardized indirect effect (β = 0.093, SE = 0.021, 95% CI [0.055, 0.139], *p* < 0.001). For Saudi nurses, AI attitude negatively predicted presenteeism (β = −0.318, CR = −5.839, *p* < 0.001) and positively predicted work engagement (β = 0.406, CR = 7.453, *p* < 0.001), while presenteeism negatively predicted work engagement (β = −0.274, CR = −5.201, *p* < 0.001), producing a significant standardized indirect effect (β = 0.087, SE = 0.021, 95% CI [0.051, 0.135], *p* < 0.001).

**TABLE 9:** Group-specific structural path estimates (Egyptian vs. Saudi nurses): unstandardized and standardized.

Path	Egyptian B	SE	CR	Egyptian β	Saudi B	SE	CR	Saudi β
AI attitude → Presenteeism	−0.566	0.069	−8.230***	−0.406***	−0.450	0.077	−5.839***	−0.318***
Presenteeism → Work engagement	−0.133	0.028	−4.740***	−0.228***	−0.149	0.029	−5.201***	−0.274***
AI attitude → Work engagement (direct)	0.376	0.041	9.094***	0.461***	0.313	.042	7.453***	0.406***
AI attitude → Work engagement (indirect)	0.075	–	–	0.093***	0.067	–	–	0.087***

B = unstandardized regression weight; SE and CR (critical ratio) refer to the unstandardized estimate (B); β = standardized coefficient. Indirect-effect SE/CR are not reported, as significance for indirect (mediated) effects was assessed via bias-corrected bootstrapped confidence intervals rather than the CR statistic.

***p < 0.001.

The group-specific structural models are illustrated in Figures 2, 3, corresponding to the Egyptian and Saudi subsamples, respectively.

**FIGURE 2:**
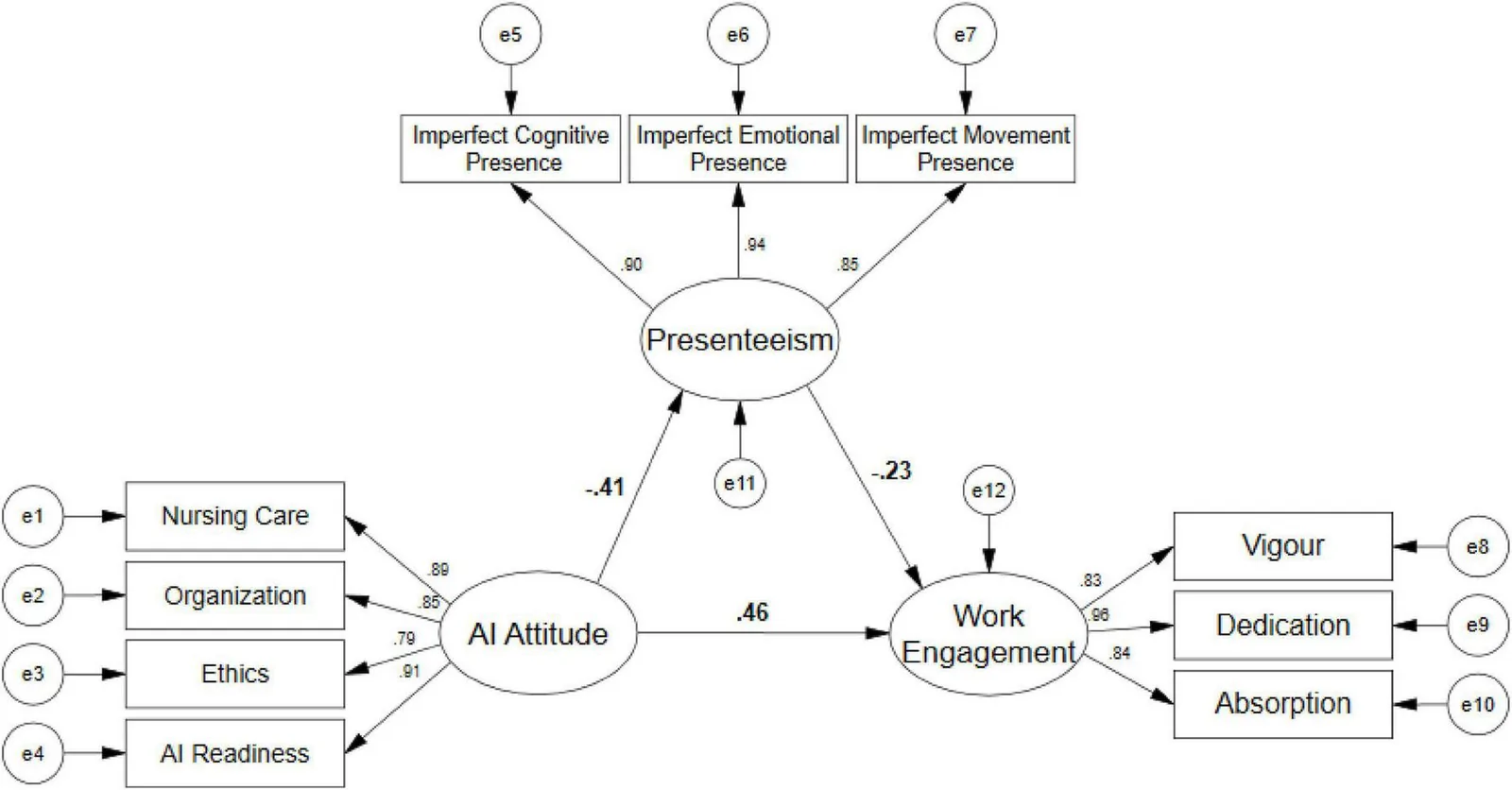
Standardized structural equation model of AI attitude, presenteeism, and work engagement among Egyptian nurses.

**FIGURE 3:**
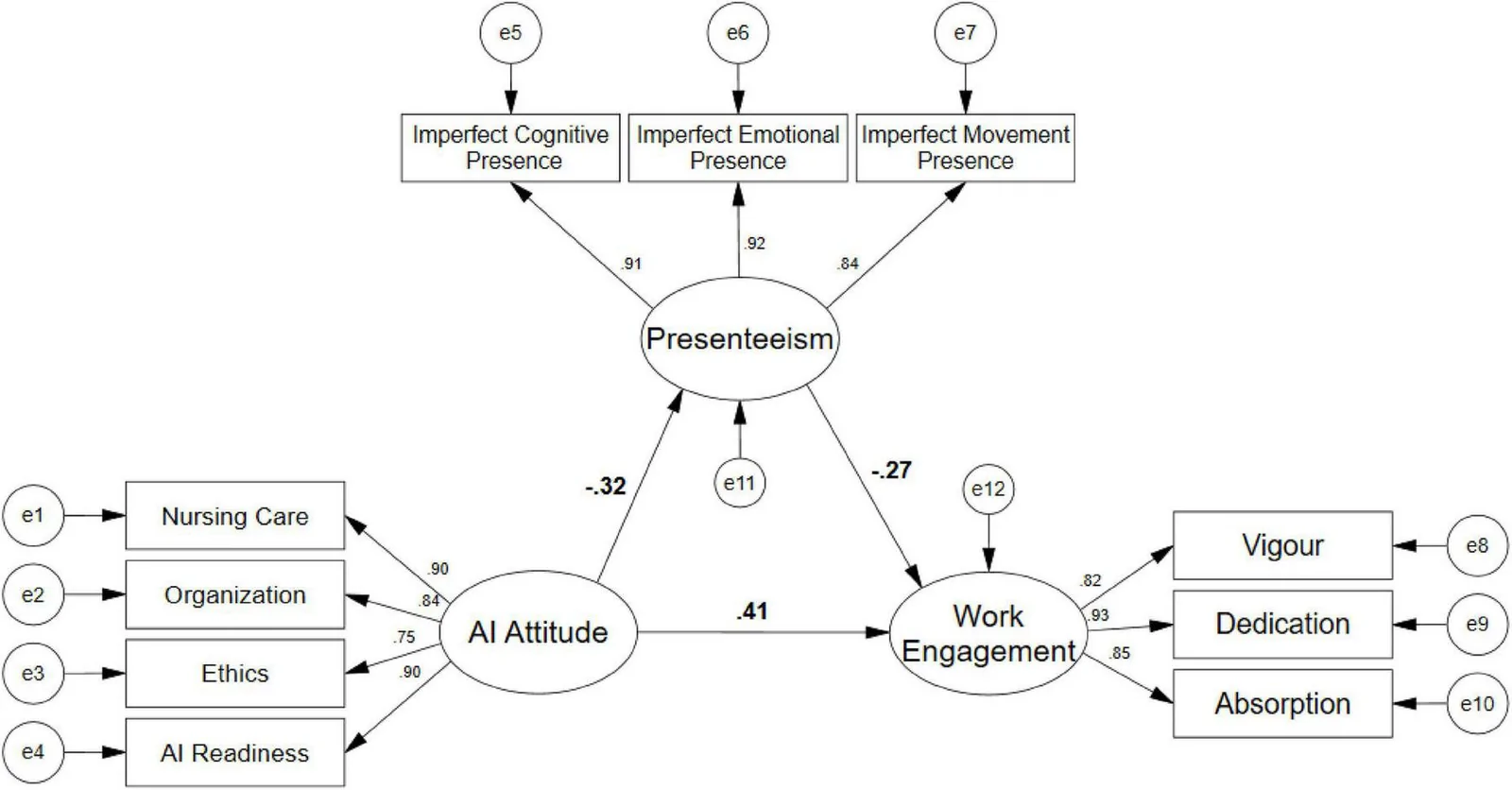
Standardized structural equation model of AI attitude, presenteeism, and work engagement among Saudi nurses.

Finally, the proportion of variance explained by the model differed modestly by context. Squared multiple correlations for the Saudi subgroup, presented in Table 10, indicated that AI attitude accounted for approximately 10.1% of the variance in presenteeism (R^2^ = 0.101) and, together with presenteeism, approximately 31.1% of the variance in work engagement (R^2^ = 0.311) among Saudi nurses. Indicator-level reliability was also strong within this subgroup, with squared multiple correlations for individual subscales ranging from 0.555 (Ethics) to 0.859 (Dedication), mirroring the pattern of loadings observed in the pooled sample.

**TABLE 10:** Squared multiple correlations (variance explained) for latent and observed variables.

Variable	Total sample R^2^	Saudi subgroup R^2^
Presenteeism (latent)	0.134	0.101
Work engagement (latent)	0.332	0.311
NC	0.806	0.814
ORG	0.717	0.707
ETH	0.594	0.555
AIR	0.821	0.810
PCOG	0.826	0.835
PEMO	0.867	0.856
PMOV	0.719	0.711
VIG	0.679	0.664
DED	0.895	0.859
ABS	0.711	0.728

Taken together, these results support all four hypothesized pathways: AI attitude was significantly and negatively associated with presenteeism, presenteeism was significantly and negatively associated with work engagement, AI attitude was significantly and positively associated with work engagement both directly and indirectly through presenteeism, and this overall pattern of relationships was invariant across Egyptian and Saudi nurses, with only minor differences in the relative magnitude of specific paths between the two national contexts.

## 4 Discussion

The present findings provide robust support for a mediation model situating presenteeism between nurses’attitudes toward AI and their work engagement. AI attitude exerted both a direct positive effect on engagement and an indirect effect operating through reduced presenteeism, confirming complementary partial mediation. This pattern is consistent with favorable AI attitudes being associated with higher engagement both directly and through an indirect association involving lower presenteeism that otherwise erodes engagement. The moderate-to-strong standardized loadings and acceptable fit indices indicate that the three constructs are empirically distinct yet theoretically interlocking, consistent with a health-impairment and motivational dual-pathway process. These results are consistent with the interpretation that nurses’psychological orientation toward emerging technology may be a meaningful occupational resource associated with how nurses function and remain engaged within increasingly digitalized clinical environments, though this cannot be confirmed causally from the present design.

This interpretation warrants appropriate caution. Although the audited model reveals a statistically significant negative association between AI attitude and presenteeism, this finding derives from cross-sectional, self-reported data, and alternative explanations–including common-method bias, residual measurement error, or reverse causal ordering (whereby lower presenteeism may precede, rather than follow, the development of favorable AI attitudes)–cannot be ruled out. Accordingly, AI attitude is characterized as a plausible rather than a confirmed protective resource within the Job Demands-Resources framework. The present findings should not be interpreted as evidence that favorable AI attitudes causally reduce presenteeism, nor should they be construed as a general endorsement of AI’s beneficial role in nursing practice. Establishing the directionality and robustness of this relationship will require longitudinal or experimental designs.

One of the most striking findings of this study is that the relationship between nurses’AI attitudes and work engagement was not merely direct, but also operated through a partial mediating pathway involving presenteeism. This pattern of associations is consistent with the possibility that negative attitudes toward AI do not simply co-occur with lower engagement, but also relate to a state of cognitive and psychological depletion associated with attending work despite diminished functional capacity, although this sequence cannot be established from cross-sectional data. Equally notable was the stability of this pattern across the Egyptian and Saudi subsamples despite differing organizational and cultural contexts. Descriptively, the AI attitude–presenteeism coefficient was numerically larger among Egyptian nurses, and the presenteeism–engagement coefficient was numerically larger among Saudi nurses; however, since the formal invariance test did not indicate statistically significant between-group differences, these numerical variations are not interpreted substantively and are not attributed to differing sources of occupational strain without further statistical support.

These findings align closely with prior literature linking positive AI attitudes to job performance, innovative work behavior, and clinical reasoning, all behaviorally proximal to engagement (Atalla et al., 2024, 2025; Morales-García et al., 2025). The negative AI attitude–presenteeism pathway corroborates evidence that technological anxiety and perceived threat deplete psychological resources (Arab et al., 2025; El Arab et al., 2025; Guo et al., 2025; Basha et al., 2025; Khairy et al., 2024). The presenteeism–engagement association reinforces documented links between impaired functional presence and reduced vigor and vigilance (Choi et al., 2024; Khoza et al., 2024). Collectively, results extend the Job Demands-Resources model (Bakker et al., 2022; Demerouti and Bakker, 2011) by showing associational patterns consistent with AI attitudes being interpreted as dual-pathway resources/demands, in line with conservation-of-resources reasoning (Xanthopoulou et al., 2007; Manresa et al., 2024).

These associational findings carry potential implications for nursing management and healthcare policy, pending confirmation through longitudinal or intervention research. Hospital administrators might consider structured AI literacy training and transparent communication regarding technology’s role in clinical practice, given that more favorable attitudes were associated with lower presenteeism and higher engagement in this sample. Organizational leaders could monitor early indicators of technology-related strain, integrating occupational health screening alongside digital transformation initiatives, particularly during Vision 2030-driven modernization in Saudi Arabia and parallel digitalization efforts in Egypt.

Human resource policies might also consider presenteeism-related strategies–flexible scheduling, adequate staffing, and psychological support–as complementary considerations alongside technology-acceptance programs. Because invariance testing confirmed equivalent structural relationships across national contexts, any interventions grounded in this model would require empirical testing before assuming transferability across similar Middle Eastern healthcare systems, with contextual calibration addressing culturally specific stressors and resource availability remaining advisable.

Practically, hospital nursing departments could pilot brief, unit-based AI-literacy workshops paired with pre/post presenteeism and engagement audits to test whether attitude change translates into measurable reductions in sickness presenteeism. From a research standpoint, longitudinal designs tracking nurses across AI system rollouts would clarify whether attitude change precedes or follows changes in presenteeism, addressing the directionality of this relationship. Future studies should also test digital self-efficacy and organizational AI-readiness as moderators, which would clarify for which nurses and under which conditions positive AI attitudes are most strongly associated with lower presenteeism.

Several limitations warrant consideration when interpreting these results. The cross-sectional design precludes causal inference regarding the directional and temporal relationships among AI attitudes, presenteeism, and engagement. Reliance on convenience sampling and self-report measures introduces potential selection and common-method bias; Harman’s single-factor test provided only a preliminary diagnostic and does not establish that common-method variance was not a contributing factor, and this limitation is acknowledged as a remaining alternative explanation for the reported associations. Additionally, the use of raw, unweighted PSN subscale sums, rather than the original scale’s differentially weighted 0–100 transformation, is acknowledged as a limitation; consequently, absolute presenteeism scores should not be interpreted against the original scale’s clinical benchmarks. Additionally, participants were nested within 12 hospitals; clustering effects were not formally examined, and observations were treated as independent – a limitation future studies should address via multilevel modeling. The study was restricted to hospital nurses in Saudi Arabia and Egypt, limiting generalizability to other healthcare professions, care settings, or cultural contexts. Additionally, unmeasured confounders – such as organizational AI implementation maturity, leadership support, or individual technology exposure – may have influenced observed relationships.

A further limitation concerns the operationalization of AI attitudes: because the AIASN assesses generalized psychological attitudes toward AI rather than documented interaction with specific AI systems, the study cannot establish whether participants had direct hands-on experience with AI tools, which technologies were involved, or whether such use was voluntary or supported by formal training; consequently, references throughout this manuscript to AI-integrated clinical work or human-AI collaboration should be interpreted as reflecting nurses’psychological orientation rather than empirically verified behavioral engagement with these technologies. Finally, although model fit indices were generally acceptable, the elevated χ^2^/df ratio and RMSEA, partly attributable to large sample size, suggest that alternative or more parsimonious specifications should be explored in future replications.

Future research should employ longitudinal or experimental designs to establish temporal precedence and causal directionality among AI attitudes, presenteeism, and engagement. Expanding samples to include additional healthcare professions, countries, and care settings would strengthen generalizability and allow further cross-cultural invariance testing. Incorporating objective indicators of AI exposure, organizational readiness, and performance metrics alongside self-report measures would reduce common-method concerns. Future studies might also examine additional mediators or moderators–such as digital self-efficacy, organizational support, or leadership style–to refine the proposed dual-pathway mechanism. Qualitative approaches could enrich understanding of how nurses subjectively experience AI-related presenteeism. Finally, intervention studies testing AI literacy or resource-based programs would provide stronger evidence regarding the practical effectiveness of strategies derived from this study’s theoretical model.

## 5 Conclusion

This study provides evidence that nurses’attitudes toward artificial intelligence are significantly associated with their work engagement both directly and indirectly through presenteeism, a pattern consistent across Egyptian and Saudi contexts. Interpreted within the Job Demands-Resources model, findings are consistent with favorable AI attitudes functioning as motivational resources related to vigor, dedication, and absorption, while unfavorable attitudes may be interpreted as activating a health-impairment pathway associated with presenteeism and, in turn, lower engagement, a causal sequence that longitudinal or experimental designs would be needed to confirm. The robustness of the measurement and structural models, together with confirmed multi-group invariance across these two national samples, strengthens confidence in the consistency of this associational pattern, though generalizability to other cultural or occupational contexts remains to be established. These associational findings underscore the potential relevance of positive technological attitudes and presenteeism management for nurse engagement amid accelerating healthcare digitalization, offering a theoretical foundation and preliminary practical considerations for future research and organizational intervention design.

## Data Availability

The raw data supporting the conclusions of this article will be made available by the authors, without undue reservation.
